# Human Serum Albumin: 3D Insight on Protein Hydration

**DOI:** 10.3390/ijms262412192

**Published:** 2025-12-18

**Authors:** Marina V. Fedotova, Sergey E. Kruchinin

**Affiliations:** G.A. Krestov Institute of Solution Chemistry, The Russian Academy of Sciences, Akademicheskaya St., 1, 153045 Ivanovo, Russia

**Keywords:** protein, human serum albumin, hydration structure, 3D-RISM integral equation method

## Abstract

Human serum albumin (HSA) is one of the main proteins in human blood plasma and serves as a molecular “taxi” transporting various compounds, including organic compounds, drugs, metal ions, etc., through the circulatory system throughout the human body. As with any other proteins, HSA hydration plays an important role in maintaining its structure and functioning as well as influencing its ability to bind to ligands. This contribution presents, for the first time, a generalized picture of hydration of this biomacromolecule obtained within the framework of the 3D-RISM (three-dimensional Reference Interaction Site Model) theory of solvation. Based on 3D isodensity maps and structural parameters (hydration numbers, hydration layer thickness, fraction of hydrogen bonds, SASA, etc.), the most probable model of HSA hydration structure was reconstructed. With the description of HSA hydration, two important issues were also addressed in detail. The first is the correct determination of the hydration layer thickness, a common problem in protein science. The second is the possible state and behavior of hydration water in HSA–ligand binding. The presented results provide a deeper understanding of the relationship between solvent and HSA, which brings new knowledge to the understanding of protein hydration.

## 1. Introduction

Hydration plays a key role in controlling the stability of protein structure, managing protein functions, and influencing protein binding to the ligand. In addition, the internal water molecules buried within proteins can be considered as “internal ligands” [1], contributing to these properties. Therefore, knowledge of the features of protein hydration and behavior of water in its hydration shell is crucial for understanding the biological processes occurring in living organisms, as well as the role of water in hydrated biosystems. However, until now, obtaining a generalized picture of the protein hydration and hydration layer itself as well as analyzing this hydration water is challenging for both experimental and in silico methods. In particular, although various experimental techniques (different types of X-ray diffraction, neutron scattering, NMR measurements, IR and THz spectroscopies, dielectric relaxation, etc.) allow one to study the structure and behavior of water in the vicinity of biomacromolecules (see, for example, review [2]), none of them can provide a holistic view of their hydration environment, which is often disordered or dynamically averaged [3]. On the other hand, for non-empirical methods including MD simulations or theoretical approaches based on the classical (and classical site) density functional theory, the large number of atoms in biomolecular systems and the diversity of interactions involved create significant difficulty in treating many-body phenomena, requiring significant computational costs. An attractive way to overcome experimental and simulation challenges is to use the 3D-RISM (three-dimensional Reference Interaction Site Model) approach [4,5]—an integral equation method of the statistical theory of liquids. This approach is known to be a powerful technique, providing a rather accurate 3D molecular picture of solute hydration, and, therefore, in recent decades, has become very popular for studying various biocompounds including proteins [1,6,7,8,9,10,11,12,13,14,15,16,17,18,19,20,21,22,23,24,25]. Using an atomic model of water and spatial distribution functions (SDFs), this method can predict the distribution of water quite accurately while maintaining a relatively low computational cost [26].

In this contribution, we apply the 3D-RISM method to present a generalized picture of the protein hydration using the most abundant protein in blood plasma, human serum albumin (HSA, PDB ID: 1AO6), as an example. We study the extent of hydration associated with the structural integrity of this protein.

### Brief Characteristics of HSA

HSA is a well-studied plasma protein, also known as the “multifaceted enzyme” [27]. Due to its extraordinary ligand-binding properties, it becomes a key player in different bioprocesses in living organisms such as transportation, distribution or metabolism of variety of compounds including as amino acids, fatty acids, vitamins, hormones, drugs, metal ions, etc. [28,29,30]. In addition, HSA is a biomarker of a set of diseases and is used in therapy in clinical practice as well as in biotechnologies, for example, as an adjuvant of cell growth and productivity or as the inner layer in special organic nanotubes for biomedical applications (for details, see [30]).

**Figure 1:**
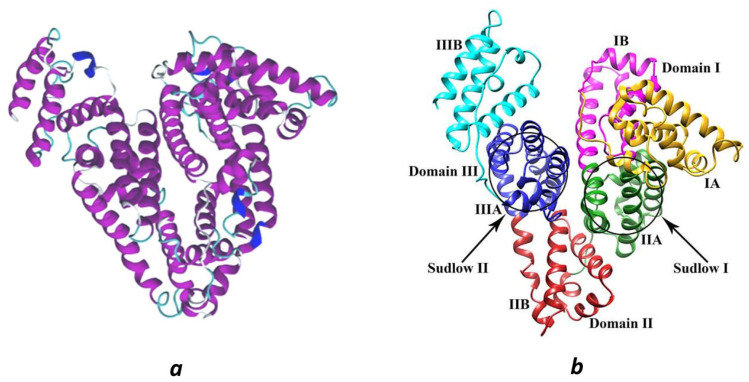
(**a**) Three-dimensional α-helical structure of HSA (PDB ID: 1AO6 [60]). The α-helices are in purple, the 3_10_-helices are in blue, the turns are in light blue, and the random coils are in white. (**b**) General molecular structure of HSA with the various color representation of domains, subdomains, and Sudlow’s binding sites I and II. Reprinted from [37], Copyright (2017), with permission from Elsevier.

HSA is a highly soluble and stable monomeric globular protein with a mass of ~66 kDa. As found in [31], its primary sequence consists of 585 amino acid residues with 17 pairs of disulfide bridges and one free cysteine (Cys34) in a single polypeptide chain. Several charged amino acid residues (Arg, Lys, Glu, and Asp) in this chain give a total negative charge to HSA at physiological pH and provide it with hydrophilic properties [32]. X-ray crystallography data showed that the HSA molecule has three structurally similar α-helical domains [33,34] and is organized into the form of an asymmetric “heart” [33] (Figure 1a). Each of these homologous domains (I, II, and III) is known to consist of two separate helical subdomains (A and B) (Figure 1b), with cross-linking stabilized by the above-mentioned 17 disulfide bonds [35]. Due to the “heart-shaped” arrangement of domains I–II and II–III, HSA has multiple sites capable of binding different classes of ligands, both endogenous and exogenous molecules (see, for instance, [30]). However, only two high-affinity ligand binding regions are in the HSA structure which are located in the hydrophobic pockets of subdomains IIA and IIIA, also called Sudlow site I and Sudlow site II [36,37] (Figure 1b).

There are studies on the HSA hydration state and the analysis of hydration water around this protein performed by different methods including IR spectroscopy [38,39,40,41,42,43], NMR [44,45], fluorescence [40,46,47], light scattering [40,47], isothermal calorimetry [41], circular dichroism [40,46,47], high-precision densitometry [48], solvation dynamics [49,50], and THz spectroscopy [51,52,53] (note that here we do not consider articles on the has–ligand binding, as this is not the subject of this contribution). In particular, attempts were made to determine HSA hydration level under different conditions, the thickness of its hydration layer and its hydration number, as well as the dynamics of water in its hydration shell and the strength of its interactions with the hydrating water. However, since these methods have different physical bases and measurement scales, the data obtained by them can differ significantly from each other. In addition, another reason for the discrepancies is the concept of the hydration shell used (whether it consists of a monolayer or several layers of water) [49,54]. We will consider this issue in detail below.

The calculations of HSA hydration parameters in the framework of 3D-RISM theory [55,56,57] were carried out in infinite diluted aqueous solution at ambient conditions using our own program code, rism3d, [58] and the modified version of the SPC/E water model (MSPC/E) [59]. The ligand-free crystal structure of the HSA protein molecule was taken from the Protein Data Bank (PDB ID: 1AO6 [60]). Intramolecular protein disulfide bonds were added according to the data from [61]. The choice between protonated and deprotonated forms of amino acid residues was made based on literature data and the results of the H++ web service [62]. The parameters describing the interaction of HSA atoms with the solvent were taken from the AMBER ff14SB parameter set [63,64]. The final structure of the HSA protein is shown in Figure 1a.

## 2. Results

### 2.1. Description of HSA Hydration: SDFs

SDFs allow for a visual representation of the distribution of solvent around a solute using isodensity surfaces. In particular, this makes it possible to determine the areas of the most probable location of the corresponding solvent sites in the vicinity of the compound. In our case, the presence of these regions is the result between the interactions of the corresponding water sites and the relevant functional groups of the protein. Isodensity surface analysis, therefore, is an attractive way for identifying hydrophilic and hydrophobic regions in proteins, as well as their hydrogen bonds with solvent, and thus provides qualitative and quantitative characteristics of biomolecule hydration.

**Figure 2:**
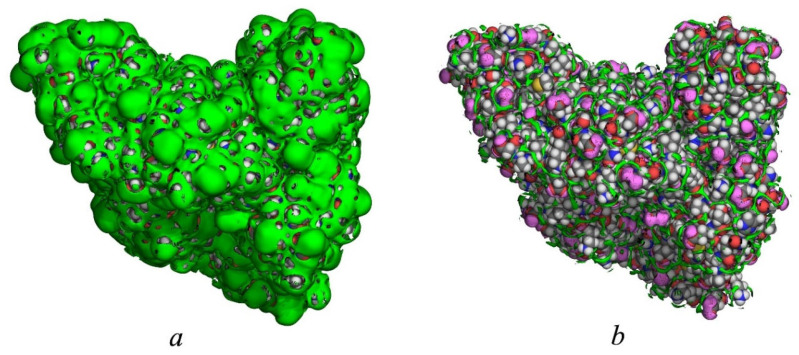
Isosurface representations of the SDFs of solvent around the HSA. (**a**) The surface for water oxygens (green regions) at *g*_Ow_(**r**) = 2. (**b**) The surface for water oxygens at *g*_Ow_(**r**) = 4 (green regions) and water hydrogens at *g*_Hw_(**r**) = 3 (pink regions). The protein atoms are colored in gray for C, in white for H, in red for O, in blue for N, and in yellow for S.

Figure 2 shows isosurface representations of SDFs for water’s oxygen and hydrogen around HSA at different thresholds. With g_Ow_(**r**) = 2 (Figure 2a), the isosurface of oxygen atoms almost completely surrounds the biomolecule, demonstrating a well-defined hydration shell. This reflects a significant interaction between HSA and the solvent. Given that g_Ow_(**r**) = 1 in the bulk phase, g_Ow_(**r**) = 2 (Figure 2a) means water molecules are distributed twice as probable as in the bulk phase. In other words, the isosurface of g_Ow_(**r**) = 2 represents the area of solvent distribution around HSA where the probability density of finding a water molecule is two times higher than in the bulk solvent. This is consistent with experimental data (see, for example, [3,65,66,67]), which shows that the first hydration layer of proteins has higher density than the bulk solvent.

Figure 2b contains two distributions of water oxygens and hydrogens represented by isosurfaces at g_Ow_(**r**) = 4 and g_Hw_(**r**) = 3, respectively. These isosurfaces indicate the hydration shell localized around the hydrophilic regions of the HSA surface. As found, the number of tightly bound solvent molecules was close to the number of solvent molecules accessible to polar atoms on the HSA surface. From Figure 2b one can see a well-developed network of H-bonds, where the oxygen distribution is localized predominantly near the groups capable of acting as hydrogen bond donors (such as hydroxyl or amino groups), while the hydrogen distribution is found, mainly, in the vicinity of the groups capable of acting as hydrogen bond acceptors (hydroxyl or carboxylate groups). This fact is connected to the high directional structure of the hydrogen bond. The result obtained is evidence of strong H-bonds between surrounding water molecules from the first hydration layer and polar sites on HSA. This has been discussed many times in studies of other proteins, including those using the 3D-RISM method [1,6,8,9,68,69].

### 2.2. Description of HSA Hydration: Total Hydration Number, and Thickness of the Hydration Shell

The molecular mass of proteins ranges from several units/tens of kDa (small proteins) to tens of thousands of kDa (large proteins). Most proteins are typically between 5.5 and 220 kDa. Today, it is known that only a few hundred water molecules per small protein or several thousand water molecules per mid-size (medium-sized) protein are sufficient to form one hydration shell around the protein (see, for instance, [70,71,72,73]). Since HSA is a mid-size protein, its total hydration number should be in the corresponding range. 3D-RISM calculations using Equation (1) (Section 4), according to the algorithm described by us in [1], give its total hydration number of 2399.1 with a protein hydration shell thickness of 0.4 nm. In addition, 65% of these hydration water molecules are H-bonded to the protein. This means that the HSA hydration layer also contains water without hydrogen bonds to the protein, which is also confirmed by IR spectroscopy data [42]. The obtained results indicate the formation of an extensive H-bonded network between water and specific sites on the HSA. This fact has been noted repeatedly in the literature when analyzing factors (including H-bonding) affecting the structural stability of globular proteins (see, for example, [54,74]).

At the same time, albumin hydration numbers, as well as consideration of the extension of protein hydration effects presented in the literature, are rather contradictory. Many authors of both experimental and computational studies have noted this fact. For example, a number of studies claim an enormous hydration number of albumin (tens of thousands of water molecules) [42,52]. Therefore, the question of the hydration number and thickness of the albumin hydration shell requires a separate discussion.

#### Thickness of the Hydration Shell: Size Does Matter

The question of the spatial range of protein hydration is a common problem in the study of biomacromolecules and has been repeatedly raised in the literature (see, for example, recent reviews [2,75,76]). It has both physical (qualitative) and structural–geometric (quantitative) foundations. Both are interrelated. The former determines how far from the protein surface the disturbance in the physicochemical properties of the solvent, caused by the interactions of proteins with their aqueous surroundings, extends. Depending on the method used to determine this spatial range, the quantitative parameters of protein hydration may vary. This situation is illustrated in Table 1, which contains data on albumins. Since literature data on structural parameters of HSA hydration are scarce, data on BSA (bovine serum albumin), which has ~76% sequence homology and the same domain organization as HSA, are also included.

**Table 1:** Structural characteristics of albumin hydration.

Albumin	Hydration Number	Thickness of Hydration Layer	Method	Reference
HSA	~24,000 (inf dilution) ~10,000–6000 (diluted solutions)	−	IR spectroscopy	[42]
HSA	~3500	0.85 nm	THz and IR spectroscopies	[77]
HSA	~2399	0.4 nm	3D-RISM	This work
BSA/HSA	1422	close to one water layer	Dielectric spectroscopy	[78]
BSA	~1200	−	Dielectric spectroscopy	[79]
BSA	~1070	−	Dielectric spectroscopy	[80]
BSA	~1100	−	Small-angle neutron scattering	[81,82]
BSA	~20,000 and higher	1.5 nm	THz spectroscopy	[52]

On the other hand, the hydration number of a protein directly correlates with the thickness of its hydration shell. Therefore, it is crucial to accurately define this value [1]. The rigorous definition of the term “the thickness of protein hydration layer” is a non-trivial issue. Results obtained depend on sensitivity, accuracy, and technical details of the method used for this purpose. This leads to large discrepancies in determination of thickness of hydration layer, not only by different methods but also within the same method (see Table 1). Due to these differences, hydration shells of proteins may consist of hundreds, thousands, and even tens of thousands of water molecules. Moreover, information provided in the literature is often based on questionable interpretations of experimental data. This practice led to a number of ambiguous results, which in turn generated contradictions and confusion in the matter (see discussion in [75,76]). For instance, terahertz (THz) spectroscopy estimates the hydration layer of small proteins (such as helix bundle protein λ_6–85_ and its mutants, myoglobin, lysozyme, ubiquitin, etc.), extending more than 2 nm from their surface [51,53]. However, for medium-sized BSA (583 amino acids), this technique gives a hydration shell thickness of 1.5 nm [52]. It is obvious that the spatial range of protein hydration found in these studies is quite large and, as a result, allows for a huge number of water molecules (for BSA it ranges from 20,000 to much higher, depending on the THz frequency under consideration [52]). A comparable value (24,000 water molecules per hydration layer in infinite diluted HSA solution) is given by data obtained by the McCabe–Fisher method within the framework of the analysis of corresponding IR spectra [42]. At the same time, the author’s explanation [42] of such large hydration numbers for this mid-size protein based on the “hydration-layer overlap” hypothesis and reasoning about its possible influence on many more water molecules than just the first hydration layer is unconvincing.

The results presented above indicate that albumins have very high hydration numbers and a very extended hydration layer. However, can these proteins really interact with the solvent at such distances (several times the diameter of a water molecule)?

Meanwhile, another study using THz spectroscopy [77] revealed a layer containing ~3500 hydration water molecules extending to 0.85 nm from the protein surface (Table 1). Furthermore, the authors [77] note that this layer consists of three to four water layers, meaning that the actual layer in direct contact with the protein involves only 1167/875 solvent molecules. From Table 1 it can also be seen that other methods yield results that differ strikingly from those obtained using THz spectroscopy. In particular, the data obtained from dielectric spectroscopy show the thickness of the albumin hydration shell to be close to one water layer [78] with BSA hydration numbers of ~1070 [80], ~1200 [79], and 1422 [78]. Close to these values is the value of ~1100 extracted from SANS data [81,82]. In silico methods, such as MD simulations or the RISM approach, typically estimate a hydration layer thickness in the range of 0.3–0.4 nm to 0.8 nm [1,6,8,49]. For example, our calculations for mid-size proteins PTP1B (protein tyrosine phosphatase 1B) [6] and HSA (this study) give 0.39 nm and 0.4 nm, respectively. Thus, estimates of the thickness of protein hydration shells and protein hydration numbers presented in the literature are also very contradictory.

Nevertheless, direct structural methods, both experimental (X-ray and neutron scattering [65,66,67]) and computational (MD [79,82] and 3D-RISM [1,6,9,74]), as well as the results of this work, have shown that the strongly interacting water molecules are mainly located only in the immediate vicinity of the protein, i.e., only its first hydration layer has a higher density than the bulk solvent. Furthermore, as found in [74], with an increase in the distance from the protein, the solvent density decays with oscillations and converges towards the bulk liquid density. An important conclusion is also drawn from MD data [76], according to which, when a protein is placed in solution, the protein-induced perturbation of the solvent is short-ranged, with its properties having exponential decay lengths within one hydration shell. Then, the bulk limit for water distribution is usually reached at a distance of about 1 nm from the biomacromolecule, when the structural organization of water is preserved beyond the first hydration shell [50]. The conclusion that the thickness of the protein hydration shell is close to one water layer, following from the experimental observation [78], also confirms these results. Thus, a monolayer of water molecules around the protein can be considered as its first hydration shell, i.e., as the region containing nearest-neighbor water molecules to the protein, most of which are tightly bound to the biomacromolecule. Following [83], this can be called the minimum hydration level required for protein function, which is quite close to the amount needed to form a hydrogen bond network spanning a protein. It is this first hydration shell that primarily protects proteins against irreversible denaturing effects, for example, abiotic stresses such as salinity or low/high temperatures.

Since the hydration shell thickness depends strongly on protein shape, the way to define it using different cutoff distances (the location of the first minimum of relevant radial distribution functions) in computational studies is not a suitable technique. This is because variations in this distance can lead to significant discrepancies in hydration numbers, complicating the proper comparative description of protein hydration (see discussion in [1]). On the other hand, our idea of applying physical properties of the hydration shell (“an increased relative density of the solvent inside the layer and a reduced density at its boundaries compared to bulk water density” [1]) to this procedure leads to correct structural characteristics of compound hydration. For instance, the total hydration number of the protein BPTI (368.9) [1] calculated by us in this way actually coincides with that (368.88) obtained in an MD study [75] where the geometric criterion was used. This means that in our calculations we have the interrelated physical (qualitative) and structural–geometric (quantitative) foundations for hydration description. From this position, the number of water molecules around the protein estimated using THz spectroscopy to be at the order of several tens of thousands is not a characteristic of the protein’s first hydration layer. However, in addition to the protein’s nearest aqueous environment, other water populations may be possible at distances greater than the thickness of the first hydration shell of the protein. All of this indicates that the size of the protein hydration layer certainly matters.

### 2.3. Description of HSA Hydration: SASA

Another useful characteristic of protein hydration is the solvent-accessible surface area (SASA) which correlates with the protein hydration volume. The latter describes the volume associated with the hydration of solvent-accessible protein amino acid residues, determined by the interaction of water with charged (through electrostriction), polar (e.g., through hydrogen bonds), and non-polar (due to hydrophobic hydration) residues on the protein surface [48]. It should be noted that larger proteins expose a greater surface area to the solvent, requiring more water molecules to form their hydration shell [71].

In our case, the SASA value for HSA calculated here by the VMD program (v. 1.9.3) [84] is 29,720 Å^2^. If we compare this result with data for another mid-size protein, PTP1B (protein tyrosine phosphatase 1B, PDB ID: 2HNP), with a mass of ~37 kDa and a number of amino acid residues of 321, we see that its SASA is only ~13,800 Å^2^ [6]. This value is 2.15 times less than in the case of albumin, correlating with the number of amino acid residues, which is 1.82 times less for PTP1B compared to HSA. This result is easily explained by the fact that the SASA in an aqueous environment primarily depends on the number of exposed protein groups [83]. This can also be seen in the example of small proteins such as bovine pancreatic trypsin inhibitor (BPTI, 6.5 kDa, 58 residues), ubiquitin (UBQ, 8.58 kDa, 76 residues), and antifreeze protein (AFP, 8.39 kDa, 84 residues). As found for them in [75], the SASA values are 4093.6 Å^2^, 4459.6 Å^2^, and 4257.2 Å^2^, respectively. These values are, on average, approximately seven times lower than that of albumin, which are connected to the protein size and the number of hydrophilic residues exposed on the protein’s surface. However, it should be noted that a change in folding that buries a large number of residues would drastically alter the SASA, regardless of the residue count ratio.

Since the albumin surface is predominantly hydrophilic, meaning it is in contact with an aqueous solvent, we can say that a quite high SASA also indicates the substantial ability of HSA to interact with water and, thus, form a significant extended first hydration layer. As a result, this property ensures improved biocompatibility of albumin [85], which is crucial for drug development. Furthermore, as found in [86], the high hydrophilicity of the HSA surface contributes to an increase in its binding energy.

### 2.4. Description of HSA Hydration: Active Site Pockets

Water molecules are known to contribute to the stability of protein and its complexes and mediate its ligand binding. These processes involve both surface water (water molecules in the protein’s first hydration shell) and interface water (water molecules located in the active site pockets) (see, for example, [6,87,88]). Therefore, in addition to a pronounced hydration shell near the protein surface, the active site pockets must also be hydrated for these purposes. To determine this, we need to calculate the water distribution near the binding regions.

Despite its hydrophilic surface, HSA contains hydrophobic binding domains. These hydrophobic pockets can bind to hydrophobic compounds, allowing albumin to act as a drug delivery vehicle (see, for instance, [89,90]). As emphasized above (Figure 1), HSA has two main active site pockets with high affinity ligand binding located in subdomains IIA and IIIA, also known as Sudlow’s sites I and II [61]. Sudlow site I binds preferentially to heterocyclic anions, such as warfarin, phentylbutazone, or azapropazone. Sudlow site II has a preferential binding affinity for aromatic compounds, like ibuprofen, propofol, or diazepam [61]. As shown in a number of studies (see, for instance, [47]), subdomains IIA and IIIA are exposed to an aqueous environment.

Here, the amino acid residues of these sites were extracted from experimental literature data [33,34,35,36,61]. Sudlow’s site I is formed by the amino acid residues Tyr150, Lys195, Gln196, Arg197, Leu198, Lys199, Cys200, Ala201, Ser202, Leu203, Gln204, Phe211, Trp214, Ala215, Arg218, Leu219, Arg222, Phe223, Leu234, Leu238, Val241, His242, Cys245, Cys246, His247, Cys253, Arg257, Leu260, Ala261, Ile264, Ile290, Ala291, Glu292. At the same time, Sudlow’s site II includes Pro384, Leu387, Ile388, Asn391, Cys392, Phe395, Arg410, Tyr411, Lys414, Leu430, Val433, Cys438, Ala449, Glu450, Leu453, Val455, Arg485, and Ser489. Sudlow’s site I of HSA is a large binding pocket, where Lys195, Lys199, Arg218, Arg222, and Glu292 are at the pocket entrance. Sudlow’s site II is smaller than Sudlow’s site I and has Arg410 and Tyr411 as major polar residues forming the entrance of this binding pocket. Site I is not only larger but also more flexible than site II; therefore, suitable drugs are able to occupy different parts of the binding pocket of subdomain IIA [91].

**Figure 3:**
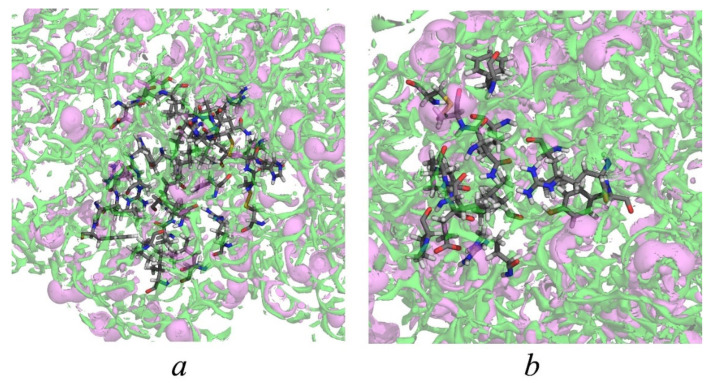
Isosurface representations of the SDFs of water oxygens at *g*_Ow_(**r**) = 4 (green) and water hydrogens *g*_Hw_(**r**) = 3 (pink) inside and in the vicinity of Sudlow’s site I (**a**) and Sudlow site II (**b**) of the HSA.

Using 3D-RISM functions, it is possible to reproduce the solvent distribution within and near corresponding active site pockets. Figure 3a,b demonstrates the isosurface representations of SDFs of water oxygens and hydrogens inside and in the vicinity of Sudlow’s sites I and II, respectively. According to these figures, there is a large amount of water around the residues. This means that both active site pockets of the protein are well-hydrated, which also follows from calculations of corresponding hydration numbers. The 3D-RISM method yields an average number of water molecules of 111.1 and 45.4 in the regions of Sudlow’s sites I and II, respectively. The number of water molecules near Sudlow’s site I is 2.5 times greater than near Sudlow’s site II, which is due to the structure and size of the first, which is almost twice as large as the second. In addition, it can be seen from the SDF of water hydrogens in the vicinity of Sudlow’s sites, *g*_Hw_(**r**) (Figure 3). According to the obtained data, H-bonds are formed preferably with polar amino acid residues of protein subdomains.

The presented results on the hydration of HSA subdomains can be supplemented by available literature data. According to a number of studies (see, for instance, [77,92,93]), protein hydration water has a larger population of strongly H-bonded water molecules compared to bulk solvent. Moreover, it is well known that the H-bonds have much longer lifetimes than those in bulk [92,93]. But it turns out that the behavior of hydration water in the active site pockets differs not only from bulk water but also from that hydrating the protein surface. This is fully applicable to Sudlow’s sites I and II of HSA. As found in [46], the residence time of hydration water (surface-bound water) is 57 ps near subdomain IIA vs. 32 ps for subdomain IIIA, i.e., the surface water molecules in site I are more ordered with longer lifetime. At the same time, bulk-type hydration is only ~0.8 ps [46], which indicates the fast water dynamics in the bulk compared to the solvent surrounding the protein. Moreover, according to the study in [46], both Sudlow’s sites are capable of forming H-bonds with solvent but only through their hydrophilic amino acids, which are more exposed to water; this is in line with our results. This fact is also supported by MD simulations [92,93], which showed that water molecules near the hydrophilic residues of the protein have a longer residence time than near hydrophobic regions on its surface.

On the other hand, the results discussed can be viewed from the perspective of protein–ligand binding. In this process, water molecules are often replaced or expelled into the bulk by drugs (see, for example, [87]) or become bridges between proteins and ligands using their own H-bonds (see, for example, [7]). Then, complex formation of proteins, including albumins, with drugs involves various types of interactions [94,95]. The longer lifetime of hydration water with its stronger hydrogen bonds at Sudlow site I, as found in [46], means that it will be more difficult for a ligand to break the hydrogen bonds between water and the protein. However, at the same time, a ligand will be able to displace non-H-bonded water molecules, which are quite abundant in the active site pocket. This conclusion can indirectly be supported by the established fact that for Sudlow site I of HSA, hydrophobic interactions, not hydrogen bonds, play a major role in controlling the affinity towards drug binding [94,95]. In turn, tightly H-bounded water can act as a bridge between the protein and the ligand during complex formation. At the same time, as found for Sudlow’s site II [46], H-bonds with water are weaker and the lifetime of hydration water is much shorter. This means that inserting a ligand into this active site pocket will be accompanied by an easier disruption of hydrogen bonds between the protein and the solvent, the replacement of the solvent by the drug, and preferential expulsion of it into the bulk. Protein–ligand binding will then involve both hydrophobic interactions and the formation of hydrogen bonds. This suggestion is consistent with results obtained for Sudlow’s site II of HSA [94,95], according to which the dominant interaction types of ligands during binding are not only hydrophobic but also H-bonding and electrostatic interactions.

Thus, the data presented allow us to conclude that the state and behavior of hydration water at Sudlow’s sites I and II will predetermine the features of ligand binding to HSA.

## 3. Discussion

The presence or absence of water in biological systems has consequences ranging from protein state to cell survival [96]. Protein–water interactions mediate protein structure, dynamics, folding, and function, as well as the structure and dynamics of hydration water surrounding the protein. Moreover, as emphasized in [46], the integrity of the protein structure is strongly correlated with the integrity of its hydration.

In this work, we carried out a 3D analysis of the hydration of medium-sized protein HSA within the framework of statistical–mechanical 3D-RISM-KH molecular theory of solvation. Based on 3D isodensitiy maps, we reconstructed the most probable model of the HSA hydration structure. The 3D-RISM results showed the presence of a well-defined hydration layer with 2399 water molecules around HSA as a monolayer of water extending approximately 0.4 nm into the bulk liquid phase. The possibility for the formation of a significant extended first hydration layer around HSA was also confirmed by a quite high SASA value. Moreover, a pronounced hydration shell near the protein’s surface assumes that a large number of water molecules (~65%) are H-bonded to HSA. Results of the study also show that Sudlow’s sites I and II of HSA, as the main active site pockets with high ligand binding affinity, are also well-hydrated and have a fraction of hydrogen-bonded water molecules. In this case, hydrogen bonds are predominantly formed with polar amino acid residues in these subdomains.

In constructing a generalized picture of HSA hydration, we addressed two important issues that we discussed in detail in our article. In particular, when describing albumin hydration, we considered the problem of correctly determining the thickness of its hydration. This problem arose due to conflicting literature data but is in fact a common one in protein science. As noted above in Section 2.2, when describing the hydration structure, a proper solution to this issue is important because the thickness of the hydration shell affects the accurate determination of protein hydration number. In this section, we emphasize the fact that protein-induced perturbation of the solvent is short-ranged and occurs only within the first hydration shell of the protein. Moreover, only the nearest-neighboring water molecules to a protein, most of which are tightly bound to it, contribute to its stability and function. We hope that our arguments and discussion will be helpful for researchers regarding this question.

The second issue we discussed was the state of water and the ligand when the latter binds to HSA. This became possible when we combined our results with literature data [46] on the residence times of hydration water in Sudlow’s sites I and II. According to our assumption, the state and behavior of hydration water at these sites predetermines the features of ligand binding to HSA. Moreover, the residence time of hydration water leads to another interesting conclusion [46]. Due to the longer residence time of hydration water, as found in [46], Sudlow’s site I is more stable than Sudlow’s site II. As a result, the authors [46] conclude that the less active site I can contribute significantly to HSA stability, while the less stable site II can contribute to HSA function. However, all these assumptions require verification, which is what we will focus on in our next study on albumin binding to ligands.

## 4. Materials and Methods

In this section, a brief description of some aspects of the 3D-RISM theory relevant to our study is presented. For more details on the methodology, readers are referred to the original literature (see, for instance, [4,5,8,21,23,55,56]).

The 3D-RISM theory provides the molecular-atom level picture of the solute hydration structure using solute–water interactions. This approach deals with the molecule–atom spatial distribution functions (SDFs), $g_{\beta }(r)$, for solvent sites *β* around the reference entire solute molecule (protein). These functions are the result of the numerical solution of two coupled equations—the 3D-RISM Ornstein–Zernike equation [56] and the 3D-Kovalenko–Hirata (KH) closure relation [55,56,57]. To calculate the SDF, one fixes a solute molecule at the origin of a local (spherical) reference frame and characterizes the local atomic densities by computing both the radial r and angular Ω=(θ,ϕ) coordinates of the solvent site position r, i.e., $g_{\beta }(r)\equiv g_{\beta }\left(r,\Omega \right)$. Since SDFs are the three-dimensional (3D) density distribution function of water atoms in a local coordinate system linked with the solute molecule, they provide a visual representation of water distribution around the solute by the isodensity surfaces.

In addition to 3D distributions of the solvent around the solute, SDFs can provide information about the characteristics of the local environment of the solute in solution. For instance, using the solute–water oxygen SDF, $g_{O_{w}}(r)$, the thickness of the solute hydration shell (the boundary of the first hydration shell) and corresponding solute hydration number can be determined. In particular, the total hydration number, *n*_tot_, of the entire solute molecule is calculated by integrating $g_{O_{w}}(r)$ over the region occupied by the solute’s first hydration shell. The number of H-bonds formed between the solute and the solvent can be determined in the same way, using the SDF $g_{H_{w}}(r)$. It should be noted that to determine the thickness of the solute’s hydration shell, we use a special procedure proposed by us in [1]. The idea of this procedure is to use two obvious properties of the hydration shell, namely, “an increased relative density of the solvent inside the layer and a reduced one at its boundaries in comparison to the density of bulk water” [1]. It follows from these properties that the minimum of the dependence of the solvent density on the distance to the compound corresponds to the boundary of its hydration layer. With this methodology, the above dependence can be defined through the derivative of the function of the number of solvent molecules on the thickness of the hydration shell, as

(1) $$n^{\prime }(r_{cut})=\frac{d}{dr_{cut}}n(r_{cut})=\frac{d}{dr_{cut}}\left(\rho_{O_{W}}\int_{V(r_{cut})}g_{O_{W}}(r)dr\right)$$

where *r*_cut_ is the distance to the protein and has the meaning of the thickness of the hydration layer, *n*(*r*_cut_) is the number of water molecules located at a distance *r* ≤ *r*_cut_ from the protein, and *V*(*r*_cut_) is the region of the first hydration shell, *V*, as a set of points located at a certain distance, *r*_cut_, from the protein surface.

The numerical solution of the 3D-RISM integral equations was performed by the MDIIS (Modified Direct Inversion in the Iterative Subspace) iterative scheme [64]. These equations were solved on a 3D grid with a spacing of 2.5 × 10^–2^ nm for each dimension and with 5 MDIIS vectors. The chosen grid size is large enough to accommodate the protein together with sufficient solvation space around them so that the obtained results are without significant numerical errors. A residual tolerance was set to 10^–6^ which was set to be enough to obtain the data with accuracy of 10^–3^.

## Data Availability

The original contributions presented in this study are included in the article. Further inquiries can be directed to the corresponding author.

## Abbreviations

The following abbreviations are used in this manuscript:

HSA	Human serum albumin
3D-RISM	Three-dimensional Reference Interaction Site Model
SDF	Spatial distribution function
SASA	solvent-accessible surface area
KH closure	Kovalenko–Hirata closure
MDIIS	Modified direct inversion in the iterative subspace

## References

[1] Kruchinin S.E., Kislinskaya E.E., Chuev G.N., Fedotova M.V. (2022). Protein 3D-hydration: A case of bovine pancreatic trypsin inhibitor. Int. J. Mol. Sci..

[2] Laage D., Elsaesser T., Hynes J.T. (2017). Water Dynamics in the Hydration Shells of Biomolecules. Chem. Rev..

[3] Svergun D.I., Richard S., Koch M.H.J., Sayers Z., Kuprin S., Zaccai G. (1998). Protein hydration in solution: Experimental observation by X-ray and neutron scattering. Proc. Natl. Acad. Sci. USA.

[4] Beglov D., Roux B. (1997). An Integral Equation to Describe the Solvation of Polar Molecules in Liquid Water. J. Phys. Chem. B.

[5] Kovalenko A., Hirata F. (1998). Three-dimensional density profiles of water in contact with a solute of arbitrary shape: A RISM approach. Chem. Phys. Lett..

[6] Kruchinin S.E., Chuev G.N., Fedotova M.V. (2023). Molecular insight on hydration of protein tyrosine phosphatase 1B and its complexes with ligands. J. Mol. Liq..

[7] Kumawat N., Tucs A., Bera S., Chuev G.N., Valiev M., Fedotova M.V., Kruchinin S.E., Tsuda K., Sljoka A., Chakraborty A. (2022). Site Density Functional Theory and Structural Bioinformatics Analysis of the SARS-CoV Spike Protein and hACE2 Complex. Molecules.

[8] Kruchinin S.E., Fedotova M.V., Kislinskaya E.E., Chuev G.N. (2023). In Silico Study of Solvation Effects in Solutions of Biomolecules: Possibilities of an Approach Based on the 3D-Distribution of Solvent Atomic Density. Biophysics.

[9] Imai T., Hiraoka R., Kovalenko A., Hirata F. (2005). Water Molecules in a Protein Cavity Detected by a Statistical−Mechanical Theory. J. Am. Chem. Soc..

[10] Phongphanphanee S., Rungrotmongkol T., Yoshida N., Hannongbua S., Hirata F. (2010). Proton transport through the influenza A M2 channel: Three-dimensional reference interaction site model study. J. Am. Chem. Soc..

[11] Chuev G.N., Valiev M., Fedotova M.V. (2012). Integral Equation Theory of Molecular Solvation Coupled with Quantum Mechanical/Molecular Mechanics Method in NWChem Package. J. Chem. Theory Comput..

[12] Eiberweiser A., Nazet A., Fedotova M.V., Kruchinin S.E., Buchner R. (2015). Hydration and Ion Binding of the Osmolyte Ectoine. J. Phys. Chem. B.

[13] Fedotova M.V., Kruchinin S.E. (2017). Hydration and Ion-Binding of Glycine Betaine: How They May Be Involved into Protection of Proteins under Abiotic Stresses. J. Mol. Liq..

[14] Dmitrieva O.A., Fedotova M.V., Buchner R. (2017). Evidence for cooperative Na^+^ and Cl^−^ binding by strongly hydrated L-proline. Phys. Chem. Chem. Phys..

[15] Fedotova M.V., Kruchinin S.E., Chuev G.N. (2017). Hydration Structure of Osmolyte TMAO: Concentration-/Pressure-induced Response. New J. Chem..

[16] Friesen S., Fedotova M.V., Kruchinin S.E., Buchner R. (2023). Hydration of the neurotransmitter γ-aminobutyric acid and its isomer α-aminobutyric acid. J. Mol. Liq..

[17] Fedotova M.V., Kruchinin S.E., Chuev G.N. (2020). Hydration features of the neurotransmitter acetylcholine. J. Mol. Liq..

[18] Sindhikara D.J., Hirata F. (2013). Analysis of Biomolecular Solvation Sites by 3D-RISM Theory. J. Phys. Chem. B.

[19] Fedotova M.V. (2019). Compatible Osmolytes—Bioprotectants: Is there a common link between their hydration and their protective action under abiotic stresses?. J. Mol. Liq..

[20] Yoshida N. (2017). Role of Solvation in Drug Design as Revealed by the Statistical Mechanics Integral Equation Theory of Liquids. J. Chem. Inf. Model..

[21] Roy D., Kovalenko A. (2021). Biomolecular Simulations with the Three-Dimensional Reference Interaction Site Model with the Kovalenko-Hirata Closure Molecular Solvation Theory. Int. J. Mol. Sci..

[22] Roy D., Kovalenko A. (2023). Multiscale Methods Framework with the 3D-RISM-KH Molecular Solvation Theory for Supramolecular Structures, Nanomaterials, and Biomolecules: Where Are We Going?. Thermo.

[23] Fedotova M.V., Chuev G.N. (2024). The Three-Dimensional Reference Interaction Site Model Approach as a Promising Tool for Studying Hydrated Viruses and Their Complexes with Ligands. Int. J. Mol. Sci..

[24] Friesen S., Kruchinin S.E., Fedotova M.V., Buchner R. (2024). Cation-Binding of Glutamate in Aqueous Solution. J. Phys. Chem. B.

[25] Kruchinin S.E., Fedotova M.V. (2025). The local hydration structure of choline-based amino acid ionic liquids in water-NaCl mixture. J. Mol. Liq..

[26] Johnson J., Case D.A., Yamazaki T., Gusarov S., Kovalenko A., Luchko T. (2016). Small molecule hydration energy and entropy from 3D-RISM. J. Phys. Condens. Matter.

[27] De Simone G., di Masi A., Ascenzi P. (2021). Serum Albumin: A Multifaced Enzyme. Int. J. Mol. Sci..

[28] Zhang G., Keita B., Craescu C.T., Miron S., de Oliveira P., Nadjo L. (2007). Polyoxometalate binding to human serum albumin: A thermodynamic and spectroscopic approach. J. Phys. Chem. B.

[29] Adachi K., Watarai H. (2006). Binding behavior of subphthalocyanine-tagged testosterone with human serum albumin at the n-hexane/water interface. Anal. Chem..

[30] Fanali G., di Masi A., Trezza V., Marino M., Fasano M., Ascenzi P. (2012). Human serum albumin: From bench to bedside. Mol. Asp. Med..

[31] Dugaiczyk A., Law S.W., Dennison O.E. (1982). Nucleotide sequence and the encoded amino acids of human serum albumin mRNA. Proc. Natl. Acad. Sci. USA.

[32] Quinlan G.J., Martin G.S., Evans T.W. (2005). Albumin: Biochemical properties and therapeutic potential. Hepatology.

[33] Sugio S., Kashima A., Mochizuki S., Noda M., Kobayashi K. (1999). Crystal structure of human serum albumin at 2.5 Å resolution. Protein Eng..

[34] He X., Carter D. (1992). Atomic structure and chemistry of human serum albumin. Nature.

[35] Carter D.C., Ho J.X. (1994). Structure of serum albumin. Adv. Protein Chem..

[36] Carter D.C., Chang B., Ho J.X., Keeling K., Krishnasami Z. (1994). Preliminary crystallographic studies of four crystal forms of serum albumin. Eur. J. Biochem..

[37] Mondal M., Lakshmi T.P., Krishna R., Sakthivel N. (2017). Molecular interaction between human serum albumin (HSA) and phloroglucinol derivative that shows selective anti-proliferative potential. J. Lumin..

[38] Murayama K., Negawa T., Hayashi T., Era S., Wu Y., Ozaki Y. (2002). Hydration study of human serum albumin in acidic pH region using two-dimensional near-infrared correlation spectroscopy. Anal. Sci..

[39] Wu Y., Czarnik-Matusewicz B., Murayama K., Ozaki Y., Murayama K. (2000). Two dimensional near-infrared spectroscopy study of human serum albumin in aqueous solutions: Using overtones and combination modes to monitor temperature dependent changes in the secondary structure. J. Phys. Chem. B.

[40] Juárez J., Alatorre-Meda M., Cambón A., Topete A., Barbosa S., Taboada P., Mosquera V. (2012). Hydration effects on the fibrillation process of a globular protein: The case of human serum albumin. Soft Matter.

[41] Sirotkin V.A., Korolev D.V., Silakova A.E. (2007). Hydration-dehydration of human serum albumin studied by isothermal calorimetry and IR spectroscopy. Russ. J. Phys. Chem. A.

[42] Dong Q., Yu C., Li L., Nie L., Zhang H., Zang H. (2019). Analysis of hydration water around human serum albumin using near-infrared spectroscopy. Int. J. Biol. Macromol..

[43] Zhang H., Liang M., Li S., Tian M., Wei X., Zhao B., Wang H., Dong Q., Zang H. (2023). Study on the secondary structure and hydration effect of human serum albumin under acidic pH and ethanol perturbation with IR/NIR spectroscopy. J. Innov. Opt. Health Sci..

[44] Baranowska H.M., Olszewski K.J. (1996). The hydration of proteins in solutions by self-diffusion coefficients NMR study. Biochim. Biophys. Acta.

[45] Pouliquen D., Gallois Y. (2001). Physicochemical properties of structured water in human albumin and gammaglobulin solutions. Biochimie.

[46] Amisha Kamal J.K., Zhao L., Zewail A.H. (2004). Ultrafast hydration dynamics in protein unfolding: Human serum albumin. Proc. Natl. Acad. Sci. USA.

[47] Mitra R.K., Sinha S.S., Pal S.K. (2007). Hydration in protein folding: Thermal unfolding/ refolding of human serum albumin. Langmuir.

[48] Sirotkin V.A., Komissarov I.A., Khadiullina A.V. (2012). Hydration of Proteins: Excess Partial Volumes of Water and Proteins. J. Phys. Chem. B.

[49] Bagchi B. (2005). Water Dynamics in the Hydration Layer around Proteins and Micelles. Chem. Rev..

[50] Chakraborty S., Sinha S.K., Bandyopadhyay S. (2007). Low-Frequency Vibrational Spectrum of Water in the Hydration Layer of a Protein:  A Molecular Dynamics Simulation Study. J. Phys. Chem. B.

[51] Born B., Kim S.J., Ebbinghaus S., Gruebele M., Havenith M. (2009). The Terahertz Dance of Water with the Proteins: The Effect of Protein Flexibility on the Dynamical Hydration Shell of Ubiquitin. Faraday Discuss..

[52] Bye J.W., Meliga S., Ferachou D., Cinque G., Zeitler J.A., Falconer R.J. (2014). Analysis of the Hydration Water around Bovine Serum Albumin Using Terahertz Coherent Synchrotron Radiation. J. Phys. Chem. A.

[53] Ebbinghaus S., Kim S.J., Heyden M., Yu X., Heugen U., Gruebele M., Leitner D.M., Havenith M. (2007). An Extended Dynamical Hydration Shell around Proteins. Proc. Natl. Acad. Sci. USA.

[54] Kuntz I.D., Kauzmann W. (1974). Hydration of Proteins and Polypeptides. Adv. Protein Chem..

[55] Kovalenko A., Hirata F. (1999). Self-consistent description of a metal–water interface by the Kohn–Sham density functional theory and the three-dimensional reference interaction site model. J. Chem. Phys..

[56] Kovalenko A., Hirata F. (2003). Three-dimensional Rism Theory for Molecular Liquids and Solid-Liquid Interfaces. Molecular Theory of Solvation.

[57] Kovalenko A., Hirata F. (1999). Potential of mean force between two molecular ions in a polar molecular solvent: A study by the three-dimensional reference interaction site model. J. Phys. Chem. B.

[58] https://github.com/sergey-kruchinin/rism3d?ysclid=mi70nhcplk19357118.

[59] Lue L., Blankschtein D. (1992). Liquid-state theory of hydrocarbon-water systems: Application to methane, ethane, and propane. J. Phys. Chem..

[60] https://www.rcsb.org/structure/1AO6.

[61] Sudlow G.D., Birkett D.J., Wade D.N. (1975). The characterization of two specific drug binding sites on HAS. Mol. Pharmacol..

[62] Anandakrishnan R., Aguilar B., Onufriev A.V. (2012). *H*++ 3.0: Automating pK prediction and the preparation of biomolecular structures for atomistic molecular modeling and simulations. Nucleic Acids Res..

[63] Maier J.A., Martinez C., Kasavajhala K., Wickstrom L., Hauser K.E., Simmerling C. (2015). ff14SB: Improving the Accuracy of Protein Side Chain and Backbone Parameters from ff99SB. J. Chem. Theory Comput..

[64] Kovalenko A., Ten-no S., Hirata F. (1999). Solution of three-dimensional reference interaction site model and hypernetted chain equations for simple point charge water by modified method of direct inversion in iterative subspace. J. Comput. Chem..

[65] McAllister C.C.W., Rudden L.S.P., Bromley E.H.C., Degiacomi M.T. (2025). The effect of hydration and dynamics on the mass density of single proteins. J. Chem. Phys..

[66] Burling F.T., Weis W.I., Flaherty K.M., Brünger A.T. (1996). Direct Observation of Protein Solvation and Discrete Disorder with Experimental Crystallographic Phases. Science.

[67] Merzel F., Smith J.C. (2002). Is the first hydration shell of lysozyme of higher density than bulk water?. Proc. Natl. Acad. Sci. USA.

[68] Sugita M., Onishi I., Irisa M., Yoshida N., Hirata F. (2021). Molecular Recognition and Self-Organization in Life Phenomena Studied by a Statistical Mechanics of Molecular Liquids, the RISM/3D-RISM Theory. Molecules.

[69] Yokogawa D., Sato H., Imai T., Sakaki S. (2009). A highly parallelizable integral equation theory for three-dimensional solvent distribution function: Application to biomolecules. J. Chem. Phys..

[70] Squire P.G., Himmel M.E. (1979). Hydrodynamics and protein hydration. Arch. Biochem. Biophys..

[71] Wilse Robinson G., Cho C.H. (1999). Role of Hydration Water in Protein Unfolding. Biophys. J..

[72] Zhou H.-X. (2001). A unified picture of protein hydration: Prediction of hydrodynamic properties from known structures. Biophys. Chem..

[73] Linse J.B., Hub J.S. (2023). Scrutinizing the protein hydration shell from molecular dynamics simulations against consensus small-angle scattering data. Commun. Chem..

[74] Imai T., Kovalenko A., Hirata F. (2004). Solvation thermodynamics of protein studied by the 3D-RISM theory. Chem. Phys. Lett..

[75] Persson F., Söderhjelm P., Halle B. (2018). The geometry of protein hydration. J. Chem. Phys..

[76] Persson F., Söderhjelm P., Halle B. (2018). The spatial range of protein hydration. J. Chem. Phys..

[77] Shiraga K., Ogawa Y., Kondo N. (2016). Hydrogen bond network of water around protein investigated with terahertz and infrared spectroscopy. Biophys. J..

[78] Yokoyama K., Kamei T., Minami H., Suzuki M. (2001). Hydration Study of Globular Proteins by Microwave Dielectric Spectroscopy. J. Phys. Chem. B.

[79] Yanase K., Arai R., Sato T. (2014). Intermolecular interactions and molecular dynamics in bovine serum albumin solutions studies by small angle x-ray scattering and dielectric relaxation spectroscopy. J. Mol. Liq..

[80] Rejou-Michel A., Henry F., de Villardi M., Delmotte M. (1985). Protein and ion hydration variation in mixed aqueous solutions: Measurement by dielectric decrement. Phys. Med. Biol..

[81] Bendedouch D., Chen S.-H. (1983). Structure and interparticle interactions of bovine serum albumin in solution studies by small-angle neutron scattering. J. Phys. Chem..

[82] Zhang F., Roosen-Runge F., Skoda M.W.A., Jacobs R.M.J., Wolf M., Callow P., Frielinghaus H., Pipich V., Prévost S., Schreiber F. (2012). Hydration and interactions in protein solutions containing concentrated electrolytes studied by small-angle scattering. Phys. Chem. Chem. Phys..

[83] Teller D. (1976). Accessible area, packing volumes and interaction surfaces of globular proteins. Nature.

[84] Humphrey W., Dalke A., Schulten K. (1996). VMD: Visual molecular dynamics. J. Mol. Graph..

[85] Meng R., Zhu H., Deng P., Li M., Ji Q., He H., Jin L., Wang B. (2023). Research progress on albumin-based hydrogels: Properties, preparation methods, types and its application for antitumor-drug delivery and tissue engineering. Front. Bioeng. Biotechnol..

[86] Moulod M., Moghaddam S. (2022). Insights from molecular dynamics simulations of albumin adsorption on hydrophilic and hydrophobic surfaces. J. Mol. Graphi. Model..

[87] Zsidó Z., Hetényi C. (2021). The role of water in ligand binding. Curr. Opin. Struct. Biol..

[88] Venkatakrishnan A.J., Ma A.K., Fonseca R., Latorraca N.R., Kelly B., Betz R.M., Asawa C., Kobilka B.K., Dror R.O. (2019). Diverse GPCRs exhibit conserved water networks for stabilization and activation. Proc. Natl. Acad. Sci. USA.

[89] Larsen M.T., Kuhlmann M., Hvam M.L., Howard K.A. (2016). Albumin-based drug delivery: Harnessing nature to cure disease. Mol. Cell. Ther..

[90] Mishra V., Heath R.J. (2021). Structural and Biochemical Features of Human Serum Albumin Essential for Eukaryotic Cell Culture. Int. J. Mol. Sci..

[91] Yamasaki K., Chuang V.T., Maruyama T., Otagiri M. (2013). Albumin-drug interaction and its clinical implication. Biochim. Biophys. Acta.

[92] Bizzarri A.R., Cannistraro S. (2002). Molecular Dynamics of Water at the Protein−Solvent Interface. J. Phys. Chem. B.

[93] Hua L., Huang X., Zhou R., Berne B.J. (2006). Dynamics of water confined in the interdomain region of a multidomain protein. J. Phys. Chem. B.

[94] Bakaeean B., Kabiri M., Iranfar H., Saberi M.R., Chamani J. (2012). Binding effect of common ions to human serum albumin in the presence of norfloxacin: Investigation with spectroscopic and zeta potential approaches. J. Solut. Chem..

[95] Anand U., Mukherjee S. (2013). Binding, unfolding and refolding dynamics of serum albumins. Biochim. Biophys. Acta.

[96] Maurer M., Oostenbrink C. (2019). Water in protein hydration and ligand recognition. J. Mol. Recognit..

